# Oxazolone-Induced Delayed Type Hypersensitivity Reaction in the Adult Yucatan Pigs. A Useful Model for Drug Development and Validation

**DOI:** 10.3390/toxins1010025

**Published:** 2009-08-21

**Authors:** Samer Nuhaily, Bassam B. Damaj, Azzam A. Maghazachi

**Affiliations:** 1 Division of Rheumatology and Immunology, University of Virginia, USA; 2 Bio-Quant, Inc., San Diego, CA, USA; 3 Department of Physiology, Faculty of Medicine, University of Oslo, Norway

**Keywords:** delayed type hypersensitivity, pigs, cyclosporine A, C-reactive protein, phorbol myristate acetate, ionomycin, chemokines, cytokines

## Abstract

The purpose of this study was to establish a model of delayed type hypersensitivity (DTH) reaction in the ear skin of large animals such as adult Yucatan pigs, which may aid in evaluating the efficacy of therapeutic modalities of newly developed anti-inflammatory drugs. The pigs were sensitized with oxazolone, re-challenged with the same irritant six days later, and dosed with either vehicle or with cyclosporine A (CsA) before and after challenge. CsA reduced the redness, inhibited the accumulation of ear fluid and inflammatory cells, as well as the release of the inflammatory mediators. Further, CsA inhibited the proliferation of T cells collected from the spleens or PBMCs of CsA-treated pigs when these cells were stimulated *in vitro* with PMA plus Ionomycin. These results indicate that pig skin can be used to evaluate modalities for the purpose of developing drugs that may be used to treat DTH in humans.

## 1. Introduction

Hypersensitivity refers to an inappropriate or exaggerated adaptive immune response which results in tissue damage. There are four main types of hypersensitivity reactions. Delayed type hypersensitivity (DTH) is a cell mediated process known as type IV. This reaction has been extensively studied in rodents, and multiple drugs for potentially inhibiting this reaction have been examined. These include cyclosporine A “CsA” [1], selectin antagonist [2], TAK-427 [3], the immunosuppressant drug periplocoside [4], *N*-acetyl-L-cysteine and *N,N’*-diacetyl-L-cystine [5], and the inhibitor of *S*-adenosyl-L-homocysteine, DHCaA [6]. However, due to the genetic differences among rodents and humans, the need to develop an *in vivo* model closer to humans is highly desirable in order to screen molecules for their immunosuppressive, anti-inflammatory and clinical applications. Pigs are genetically closer to humans and have been used for xenotransplantation purposes among porcine and baboons or humans, as well as other species [7,8,9,10,11].

CsA binds cyclophilin and inhibits the activity of the protein phosphatase calcineurin, resulting in an inhibition of T cell function [12]. It also inhibits DTH reactions in mice [1], but whether it has any effect on DTH reactions in large animals has not been extensively studied. Here, we successfully developed a DTH reaction in the ear skin of other animals, *i.e.*, Yucatan pigs, utilizing the irritant oxazolone (4-methoxymethylene-2-phenyl-2-oxazolin-5-one) which has been used to induce atopic dermatitis in mice [13], and DTH in rats [14]. Also, we examined the effects of CsA in inhibiting inflammatory and cellular events associated with DTH reactions induced by oxazolone. The purpose of developing this system is to aid pharmaceutical companies to examine the efficacy of anti-inflammatory drugs in large animals that may relate to humans more closely than rodents. Also, the system could perhaps provide information that can be utilized to develop new drugs and other procedures for potential therapy.

## 2. Materials and Methods

### 2.1. Animals

Six-month old, male Yucatan pigs (~25 kg), HLA-matched, were obtained from S&S Farms (Escondido, CA, USA). The animals were group-housed under recommendations detailed in the DHEW guide for the care and use of laboratory animals (NIH) and according to USDA regulations. All procedures were subject to IACUC review. Each animal was trained to tolerate placement in a restraining sling for up to 30 min. Training involved the daily placement of the animals in the sling for increasing periods of time over 10 days. 

### 2.2. Sensitization and challenging pigs with oxazolone

Pigs were sensitized by the epidermal injections of 2% oxazolone (1 mL total given at ~15 sites) at a localized area on the back of each pig. Test animals were challenged on day 6 by the intra-dermal injection with 1 mL of 1% oxazolone in acetone: olive oil into the left ear of each pig at about 15 sites.

### 2.3. Other procedures

Animals were lightly sedated with ketamine/xyalazine and placed in the restraining sling. CsA (Sandoz Pharmaceuticals, Princeton, NJ, USA), was administered through a feeding tube designed for foals. The utilization of oxazalone as an irritant was based on work done in the mice [15]. The procedures for dosing the pigs are summarized in Table 1. Red blood cells were lysed by resuspending the pelleted splenocytes in 155 mM NH_4_Cl, 10 mM KHCO_3 _ and 0.1 mM EDTA (Sigma-Aldrich, St. Louis, MO, USA) and incubating at room temperature for 5 min with gentle agitation. After repeating the red blood cell lysis a second time, the cells were centrifuged and resuspended at a concentration of 5 × 10^6^/mL in RPMI containing 10% fetal bovine serum. The cells were labeled by culturing with 5 µg/mL calcein AM (TefLabs, Capitol View Drive Austin, TX, USA) for 45 min. Ear biopsies were placed in HBSS (w/1.21 mM CaCl_2_, 0.493 mM MgCl_2_, 0.407 mM MgSO_4_) containing 10% FBS (HBSS/FBS). The dorsal and ventral leaflets of the ears were separated and then deposited (medial dermis aspects face down) on 1 mg/mL dispase/collagenase (Sigma-Aldrich) in HBBS/BSA. The tissue was digested at 37 ^o^C for 1 h, after which the media was collected. The media was centrifuged at 1,000× g for 10 min and the cellular contents resuspended in 200 µL of PBS. The recovered cells were placed into a well of a 96 well plate and the fluorescence at an excitation wavelength of 480 nm and emission wavelength of 530 nm measured in a Cytofluor 4000 (PerSeptive BioSystems, American laboratory Trading, Inc., East Lyme, CT, USA). The number of recovered cells from each ear was calculated from a standard curve generated from dilutions of the reserved aliquot of the labeled cell suspensions. The ears were dissected and pictures of the dermal inflammation taken. 

**Table 1:** Summary of the protocols used to sensitize and dose the pigs.

**Protocol for pigs in group 1**
Day 0: Intra-dermal sensitization with 2% oxazolone on back
Day 6, -12 hr: Bled (interleukin-2 (IL-2), interleukin-8 (IL-8), macrophage inflammatory pretin-1α (MIP-1α), and C-reactive protein (CRP) levels were measured); dosed (PO)
Day 6, 0 hr: Epi-dermal challenged with 1% oxazolone on ear
Day 6, +3 hr: Dosed (PO)
Day 6, +6 hr: Bled (IL-2, IL-8; MIP-1α, and CRP levels were measured)
Day 6-8: Pigs were sacrificed. Inflamed ears were drained, left and right ears were harvested and pictures taken.
**Protocol for pigs in group 2**
Day 0: Intra-dermal sensitization with 2% oxazolone on back
Day 6, -12 hr: Bled (IL-2, IL-8; MIP-1α, and CRP levels were measured); dosed (PO)
Day 6, 0 hr: Epi-dermal challenge with 1% oxazolone on the ears
Day 6, +2 hr: Spleens were harvested from sensitized donor pigs
Day 6, +3 hr: Dosed (PO)
Day 6, +6 hr: Bled (IL-2, IL-8; MIP-1α, and CRP levels are measured); Injected (IV) with fluorescently labeled splenocytes
Day 6, +9hr: Pigs were sacrificed. Inflamed ears were drained (IL-2 level was measured, and the number of cells were calculated). Left and right ears were dissected and pictures taken. Dermis was dissected for measuring fluorescence intensity.
Spleens were harvested (cell isolation and T cell proliferation after PMA/IONO stimulation was conducted), blood was collected (IL-2, IL-8; MIP-1α, CRP levels were measured).

### 2.4. Measurement of inflammatory mediators

The levels of porcine IL-2, IL-8, and CRP were assessed using commercial ELISA kits (Bio-Quant, Inc., San Diego, CA, USA). 

### 2.5. T cell Proliferation and IL-2 release

Peripheral blood mononuclear cells (PBMCs) and spleens were aseptically removed from the pigs, and were subjected to density gradient centrifugation on Histopaque (Sigma-Aldrich). The lymphocyte layers were washed twice and resuspended in RPMI 1640. T cell preparations were centrifuged, resuspended in 5 mL of incomplete RPMI and counted using a hemocytometer. For T cell proliferation, these cells (1 × 10^5^/mL) were incubated with 200 ng/mL each of phorbol myristate acetate (PMA) and ionomycin (IONO) (Sigma-Aldrich) in complete RPMI media. Two hundred µL of complete RPMI containing 0.86 mg/mL thiazolyl blue tetrazolium bromide (MTT, Sigma-Aldrich) was added to each well and the plates cultured for an additional 4 h. The plates were centrifuged again, the media removed and the well contents solubilized in 100% DMSO. The optical density at 560 nm of each well was determined and the average mean of the duplicate wells calculated. The background OD 560 value, determined from control wells receiving media containing no MTT, was subtracted from these values and the data normalized to that of the control wells. Similar to T cell proliferation, both PBMCs and splenocytes were removed from the animals and activated with 200 ng/mL each of PMA plus IONO in complete RPMI media. After culturing for 24-72 h, the plates were centrifuged at 1200 rpm for 10 min and the culture supernatants removed and saved. IL-2 levels were measured by ELISA assay.

### 2.6. Statistical analysis

Statistical significance between treated groups and the vehicle control was evaluated by paired *t*-test using the GraphPad Prizm program (GraphPad Software, San Diego, CA, USA). Differences between groups were considered significant at the level of *p* < 0.05. Percent inhibition was calculated as 100-values in the presence of CsA divided by values in the presence of vehicle.

## 3. Results

The results of this study are divided into two parts. The first part will present data obtained from pigs in group 1, and the second will present data obtained from pigs in group 2.

### 3.1. Effect of CsA on the ear inflammation of pigs in group 1

**Figure 1:**
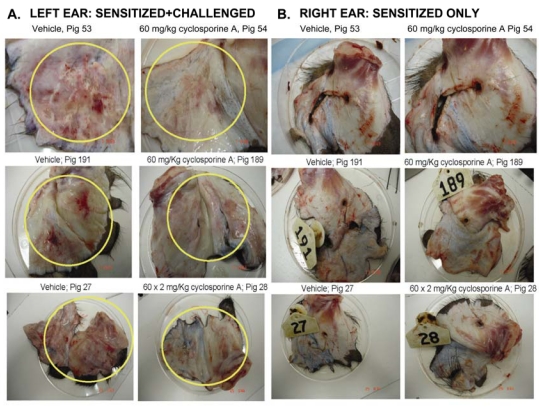
Effect of treatment with CsA on ear redness of pigs in group 1. (A) Left ears were isolated from vehicle (sensitized and challenged) pigs, or pigs dosed PO twice with 60 mg/kg CsA. Red inflammation where injections were done is shown inside yellow circles. The results from three different pigs are shown. (B) Right ears were isolated from pigs that were only sensitized but not challenged.

Results in Figure 1 indicate that in three different experiments, the ears of the vehicle pigs (# 53, 191 and 27) sensitized with oxazolone at day 0 and then challenged on day 6 with the same antigen, are highly inflamed, with clear erythema and redness (yellow circles). Figure 1 also shows an obvious reduction of these activities in pigs that received 60 mg/kg × 2 CsA (pigs #54, 189 and 28; yellow circles). Of note, 60 mg/kg CsA was chosen because its availability in the animals is lower than that observed in humans, hence a higher dose was used to ensure therapeutic levels. As a control, we also monitored inflammation in the right ears of pigs which were only sensitized but not challenged with the antigen. Notably, the right ears did not show any clear signs of inflammation. Similarly, there was no effect of CsA on the appearance of the right ears. 

### 3.2. Effect of CsA on inflammatory reactions in inflamed ears

**Figure 2:**
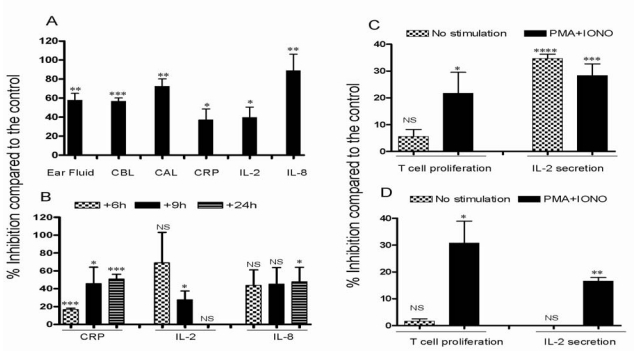
Effect of dosing with CsA on various inflammatory and cellular activities of pigs in group 1. Pigs were sensitized and challenged with oxazolone and were either treated with vehicle or with 60 mg/kg CsA twice. (A) Percent inhibition after CsA treatment as compared to the vehicle is shown as mean ± SEM collected from three pigs. Shown is the inhibition of ear fluid, number of cells before RBC lysis (CBL), cells after RBC lysis (CAL), CRP, IL-2 and IL-8 levels in sensitized ears after CsA treatment. (B) Percent inhibition of the levels of CRP, IL-2, and IL-8 in the serum 6, 9, and 24 h post dosing with CsA is shown. (C) Percent inhibition after CsA dosing of T cell proliferation and their ability to secrete IL-2 collected from PBMCs that were either unstimulated or stimulated *in vitro* with PMA plus IONO is shown. (D) Percent inhibition after CsA dosing of T cell proliferation and their ability to secrete IL-2 collected from spleens that were either unstimulated or stimulated *in vitro* with PMA plus IONO. ∗, P < 0.05, ∗∗, P < 0.01, ∗∗∗, P < 0.001, ∗∗∗∗, P < 0.0001.

Inflammation is associated with the accumulation of fluids and leukocytes [16]. We entertained the possibility that the anti-inflammatory/immunosuppressant compound CsA may have adverse effects on such accumulation. For these results and all others, percent inhibition as compared to the controls is shown. Results in Figure 2A demonstrate that ear fluid accumulating in inflamed ears was inhibited in pigs dosed with CsA, and this inhibition was significant (P < 0.01). The number of total cells (which include RBCs and WBCs, designated cell before lysis or “CBL”), accumulating in inflamed ears was also inhibited in pigs dosed with CsA, and this inhibition was statistically significant (P < 0.001). Similarly, when RBCs were lysed, the number of leukocytes accumulating in the inflamed ears (designated cells after lysis or “CAL”), was significantly reduced in the ears of pigs that received this drug (P < 0.01, Figure 2A). The levels of CRP, IL-2 or IL-8 secreted in the ears of pigs treated with CsA were also significantly reduced (P < 0.05, P < 0.05, and P < 0.01, respectively, as shown in Figure 2A). Collectively, these results strongly indicate that CsA has a profound anti-inflammatory effect toward the inflammation occurring in sensitized and challenged ears of the pigs.

Next, we examined the effect of dosing pigs with CsA on the release of the inflammatory molecules in the serum, which are responsible for the inflammatory response. Results in Figure 2B demonstrate that dosing with CsA significantly inhibited the levels of serum CRP after 6, 9 or 24 h of dosing (P < 0.001, P < 0.05, and P < 0.001, respectively). Dosing twice with this drug also reduced the level of serum IL-2. Notably, this activity did not reach statistical significance after 6 or 24 h, but the inhibition was significant after 9 h of dosing (P < 0.05, Figure 2B). Furthermore, dosing with CsA showed a trend toward reducing the accumulation of the inflammatory chemokine IL-8 in the serum of dosed pigs after 6 or 9 h, and this reduction reached statistical significance after 24 h of dosing (P < 0.05, Figure 2B). The ability CsA to affect various activities of T cells *in vitro* was next investigated. First, we examined whether PBMCs isolated from pigs sensitized and challenged with oxazolone but no CsA, or from pigs dosed as above but also treated with CsA, can be activated *in vitro*. Results shown in Figure 2C indicate that cells isolated from pigs dosed with CsA were inhibited from proliferating upon stimulation with PMA plus IONO for 72 h. Similar to PBMCs, cells isolated from the spleens of pigs dosed with CsA had significantly lower proliferation than T cells isolated from vehicle-treated pigs, upon stimulation with PMA plus IONO (P < 0.05, Figure 2D).

In addition, both spontaneous release of IL-2 (without *in vitro* stimulation) and that released upon stimulating PBMCs of CsA dosed pigs with PMA plus IONO for 48 h *in vitro* were significantly inhibited (P < 0.0001, and P < 0.001, respectively, as shown in Figure 2C). In the spleens, the inhibitory effect of CsA on IL-2 secretion was clearly noted after 48 h activation with PMA plus IONO (P < 0.01, Figure 2D).

### 3.3. Effect of CsA on the ear inflammation of pigs in group 2

**Figure 3:**
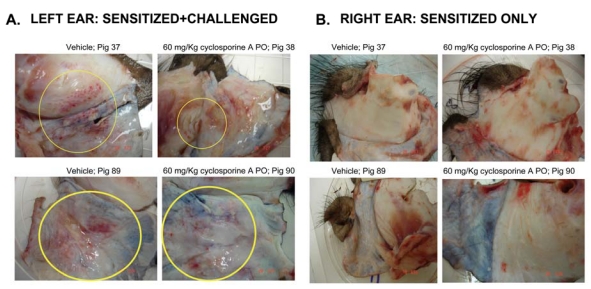
Effect of CsA treatment on ear inflammation of pigs in group 2. (A) Left ears were isolated from pigs that were sensitized and challenged with oxazolone, and were dosed with either vehicle or with 60 mg/kg CsA twice. Red inflammation where injections were done is shown inside yellow circles. (B) Right ears were isolated from pigs that were only sensitized but not challenged with oxazolone.

Pigs in group 2 provide excellent model to monitor the accumulation of inflammatory cells at sites of inflammation. There was an obvious reduction in the redness of the ears in pigs that received twice 60 mg/kg CsA (pigs #38 and 90). Similar to pigs in group 1, the inflammation in the right ears was also monitored, which did not show any clear sign of inflammation (Figure 3).

**Figure 4:**
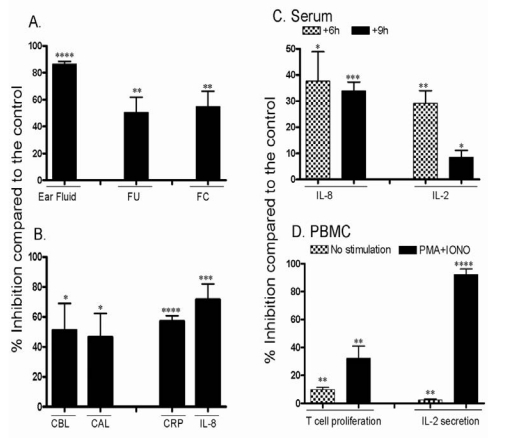
Effect of dosing with CsA on T cell activities of pigs in group 2. Pigs were sensitized and challenged with oxazolone, and were either treated with vehicle or with 60 mg/kg CsA twice. (A) Percent inhibition after CsA treatment as compared to the vehicle is shown as mean ± SEM. The inhibition of ear fluid, fluorescent units (FU), and the recovery of fluorescent cells (FC) in sensitized ears after CsA treatment is shown. (B) Shown is the percent inhibition of the number of cells before RBC lysis (CBL), cells after RBC lysis (CAL), CRP level and IL-8 levels in sensitized ears, is shown. (C) Percent inhibition after CsA dosing of serum IL-8 and IL-2 levels 6 and 9 h after dosing with CsA (24 h samples were not collected). (D) Percent inhibition after CsA dosing of T cell proliferation and their ability to secrete IL-2 collected from PBMCs that were either non-stimulated or stimulated *in vitro* with PMA plus IONO. ∗, P < 0.05, ∗∗, P < 0.01, ∗∗∗, P < 0.001, ∗∗∗∗, P < 0.0001.

In addition, we investigated the migration of cells isolated from pigs that were sensitized with the antigen. These cells were labeled with calcein AM and injected IV into vehicle pigs or pigs treated with the different compounds. Results in Figure 4A demonstrate that the amount of ear fluid (EF) recovered from pigs dosed with CsA was highly inhibited (P < 0.0001); whereas about 10 mL of EF were recovered from control pigs, less than 2 mL were obtained from pigs treated with CsA. The fluorescence units (FU) recovered from the ears of CsA-treated pigs was also inhibited when compared to the FU recovered from vehicle pigs (P < 0.01). Similarly, there was a significant inhibition of the migration of sensitized fluorescently labeled T cells (FC) into the sites of inflammation in pigs treated with CsA (P < 0.01, Figure 4A).

Similar to the approach of the first model, we investigated the possibility that CsA may ameliorate DTH by inhibiting the release of inflammatory mediators. The number of total cells (which include RBCs and WBCs; cells before lysis “CBL”), accumulating in inflamed ears was significantly reduced in pigs dosed with CsA (P < 0.05). Similarly, when RBCs were lysed, the number of leukocytes (cells after RBC lysis or “CAL”), accumulated in inflamed ears was significantly inhibited in pigs dosed with CsA (P < 0.05, Figure 4B). Further, we examined the release of the inflammatory molecules normally secreted in inflamed ears, which are responsible for the inflammatory response occurring at these sites. Dosing with CsA significantly inhibited the levels of CRP (P < 0.0001), and the inflammatory chemokine IL-8 in inflamed ears (P < 0.01), as shown in Figure 4B.

The effect of CsA on the levels of the inflammatory molecules released in the serum was next examined. Hence, the levels of IL-2 and IL-8 in the serum of pigs dosed with CsA were measured and compared to the levels of these molecules in vehicle control pigs. Results in Figure 4C demonstrate that the amount of serum IL-8 in CsA-dosed pigs was significantly inhibited 6 and 9 h post dosing (P < 0.05 and P < 0.001, respectively). Similar findings were observed when the level of serum IL-2 was measured, i.e. there was a significant reduction of IL-2 released in the serum of CsA-dosed pigs after 6 and 9 h of dosing (P < 0.01, and P < 0.05, respectively, Figure 4C).

The ability of CsA to affect various activities of T cells *in vitro* was investigated. First, we examined whether PBMCs isolated from pigs in the vehicle control or from pigs dosed with CsA are activated *in vitro*. The results show that cells isolated from pigs dosed with CsA were inhibited from proliferating, even in the absence of any stimulus (P < 0.01). Upon stimulation with PMA plus IONO for 72 h, it is clear that cells isolated from pigs treated with CsA had significantly much lower proliferation than cells isolated from vehicles (P < 0.01, Figure 4D). The ability of CsA to affect IL-2 release from PBMCs was next examined. Notably, PBMCs isolated from pigs treated with CsA had lower IL-2 release when compared to the vehicle control after 48 h *in vitro* incubation with or without stimulation with PMA plus IONO (P < 0.01, and P < 0.0001, respectively, Figure 4D). 

## 4. Discussion

DTH, a Th1 reaction, is characterized by elicitation of immune response after antigenic stimulation and then re-challenging with the same antigen. However, no response can be detected after only the sensitization step [16]. During DTH, sensitized T cells migrate toward the sites of antigenic stimulation manifesting cellular responses which include the release of Th1 cytokines such as IL-2 and IFN-γ. Other cell types such as neutrophils are also recruited to these sites, but these cells migrate earlier than T cells. One important function of these cells is secreting cytokines and chemokines which activate T cells and amplify the reaction by recruiting more T cells and antigen presenting cells to the sites of action [16]. In pigs, CD4^+^, CD8^+^ and γδ T cells were recruited into inflamed sites [17]. Other chemoattractants accumulate at sites of inflammation and also recruit various subsets of lymphocytes [18,19].

Although allergic contact dermatitis was previously established in Gottingen pigs using 2,4-dinitrofluorobenzene (DNFB) as a hapten [17], this study did not examine the effect of CsA on contact dermatitis or validate the model as a non-rodent model for drug discovery. Here we successfully established DTH in the ear skin of adult Yucatan pigs. Further, our approach of injecting fluorescently labeled sensitized cells into challenged pigs adds another important parameter of examining the infiltration of sensitized cells into inflamed sites. This is important since most DTH reactions have been previously examined in mice and rats. However, most of the small molecules which are the basis for non biological drugs that include ion channel inhibitors and G protein-coupled receptor antagonists do not cross react with mice or rats. Hence, there is a need not only to develop but also to validate such inflammation models in large non rodent animals such as the pig system. The importance of our findings rests in the relatedness of pigs to humans in terms of genetic background and immunological reactions.

Our results indicate that oxazolone successfully induced inflammation in pigs as determined by ear swelling, redness and erythema that occurred in the challenged ears, which is similar to the effect of this irritant in mice and rats [13,14,15]. Orally administered CsA robustly reduced the extent of inflammation in the challenged ears. In addition dosing pigs with this drug significantly inhibited the accumulation of ear fluid and inflammatory cells in inflamed ears. It also inhibited the accumulation and/or the release of the inflammatory mediators CRP, IL-2 and IL-8 in inflamed ears. This clearly shows an important role of CsA as an anti-inflammatory drug which inhibits the accumulation of inflammatory cells secreting inflammatory mediators such as IL-2. The fact that CsA also reduced IL-8 release at the sites of inflammation strongly indicates that neutrophils are impeded from migrating into these sites since IL-8 is a robust chemoattractant for these cells [19].

The effect of CsA goes beyond inflammatory sites (ears), as this drug also inhibited the release of IL-2, IL-8 and CRP in the serum of dosed pigs in both pig models. The systemic effects of CsA indicates that inflammatory cells that are generated after initial sensitization are found at body sites such as blood circulation and that the activity of CsA ensures that these inflammatory cells are inhibited from secreting inflammatory mediators. Not only the release of inflammatory mediators in the circulation is inhibited, but also the proliferation of T cells is impeded after dosing with CsA, which could be due to the immunosuppressive effect of CsA. This is true for PBMCs and splenic T cells. The anti-proliferative effect of CsA is more pronounced when T cells were stimulated *in vitro* with PMA plus IONO. Dosing with CsA also inhibited T cells collected from PBMCs or spleens from secreting the inflammatory cytokine IL-2. Collectively, these results show that PO dosing with CsA results in a potent anti-inflammatory effect for DTH reactions both at inflamed sites as well as at distal sites ensuring no Th1 response is taking place. However, one can not exclude the possibility of immunosuppressant effects of CsA, as has been shown in rodents [20]. It should be also mentioned that the inhibitory effects of CsA in the sensitization phase of hypersensitivity have been well documented [21,22]. Finally, our results support the notion that pigs can be used as a model to screen anti-inflammatory compounds for the purpose of generating drugs that may be used to treat DTH reactions in human. 
